# Renal microangiopathy and immune complex glomerulonephritis induced by anti-tumour agents: A case report

**DOI:** 10.1515/biol-2022-0986

**Published:** 2024-11-22

**Authors:** Li Guo, Zhen-Zhen Hao, Qian Zhang, Qiu-Ju Liu, Yan Gao, Lei Ran

**Affiliations:** Department of Nephrology, Affiliated Hospital of HeBei University, No. 212 of YuHua East Road, LianChi District, Baoding, 071000, Hebei, China; Key Laboratory of Bone Metabolism and Physiology in Chronic Kidney Disease of Hebei Province, Baoding, 071000, Hebei, China

**Keywords:** drug chemotherapy, thrombotic microvascular diseases, glomerulonephritis, focal segmental glomerulosclerosis-like lesions, case report

## Abstract

A 60-year-old woman with bilateral lower extremity oedema for several days was admitted to the hospital on 21 November 2023. Previously, after receiving rectal cancer resection in February 2023, she had been receiving drug chemotherapy, during which she had normal urinalysis and renal function. However, 10 days before admission, after the drug regimen was adjusted to tislelizumab + fruquintinib, she developed bilateral lower extremity oedema with foamy urine; this was later extended to facial oedema. After a histologic examination of renal biopsy, it was judged as drug-induced glomerular microangiopathy (GMA) with focal segmental glomerulosclerosis-like lesions accompanied by immune complex-mediated glomerulonephritis. The condition was controlled by stopping the anti-tumour drug, lowering glucose with linagliptin, and providing renal protection with Nephritis Rehabilitation Tablets, and the patient recovered well at the follow-up visit after 6 months. This case may be GMA induced by tislelizumab or fruquintinib and was examined in this study.

## 1 Introduction

New anti-cancer drugs have significantly improved the survival time of patients with cancer. Concurrently, the kidney damage caused by these drugs and its adverse impact on patient prognosis has been gaining increasing attention [1]. Traditional chemotherapy drugs primarily cause kidney damage through direct toxicity to the renal tubules, whereas new anti-cancer drugs can cause various types of kidney damage, including proteinuria, acute tubular necrosis, thrombotic microangiopathy (TMA), interstitial nephritis, and glomerular diseases [2,3]. Among them, TMA is a group of diseases characterised by endothelial injury leading to microvascular thrombosis and ischaemia. Depending on the differences in pathogenesis, clinical manifestations, and prognostic characteristics, TMA induced by anti-cancer drugs is divided into type I TMA, which is related to the direct endothelial toxicity of chemotherapy drugs, and type II TMA, caused by anti-vascular endothelial growth factor (VEGF) drugs [4–6]. Mitomycin C and gemcitabine are among the most common anti-cancer drugs that cause type I TMA. The most common drugs causing type II TMA are anti-VEGF drugs, which are divided into two categories: one targets VEGF, blocking its binding to VEGF receptors (VEGFR), such as bevacizumab; the other inhibits the intracellular domain of the VEGFR’s tyrosine kinase, such as sunitinib. VEGF plays a crucial role in maintaining the normal function of glomerular endothelial cells and the integrity of the basement membrane, promoting endothelial cell proliferation, differentiation, and survival, as well as mediating endothelial-dependent vasodilation. VEGF nourishing the glomerular endothelial cells is mainly synthesised by podocytes, as well as coming from the bloodstream. Blocking the binding of VEGF to its receptor and inhibiting the synthesis of VEGF by podocytes can damage glomerular endothelial cells, leading to the occurrence of TMA. A study by Vigneau et al. [7] reported that among 22 patients with renal adverse reactions after anti-VEGF treatment who underwent renal biopsy, 21 had TMA. This type of TMA can occur at any time during anti-VEGF treatment, is independent of drug dosage, often lacks systemic manifestations, and causes mainly renal damage, and the lesions are limited to the glomeruli. Anti-VEGF treatment can lead to a special type of glomerular microvascular endothelial cell lesion, which is sometimes known as “glomerular microangiopathy (GMA)” [8]. Once kidney damage occurs during cancer treatment, it not only causes harm to the body but can also disrupt the progression of cancer treatment plans, affecting the efficacy of anti-cancer treatment and patient prognosis. Therefore, kidney damage caused by anti-cancer drugs needs to be detected early and treated promptly.

In the field of cancer treatment, the combination of immunotherapy and targeted therapy provides new treatment strategies for patients. Tislelizumab is a humanised monoclonal antibody that targets the programmed death ligand-1 (PD-L1). By blocking the interaction between PD-L1 and programmed death receptor-1 (PD-1) receptors, tislelizumab can enhance the immune system’s ability to recognise and eliminate tumour cells, demonstrating significant efficacy in the treatment of various types of cancer [9]. Fruquintinib, as a quinazoline small-molecule angiogenesis inhibitor, primarily acts on the VEGFR kinase family (VEGFR1, 2, and 3). It effectively inhibits the phosphorylation of VEGFR on vascular endothelial cell surfaces and downstream signal transduction, thereby inhibiting the proliferation, migration, and tube formation of vascular endothelial cells, blocking the formation of new tumour blood vessels, and thus inhibiting tumour growth [10]. However, the combined use of these drugs may bring new challenges and risks. This case report describes a 60-year-old female patient who developed kidney-limited TMA after combined treatment with tislelizumab and fruquintinib. Through this case, we explore the potential risks of the combined treatment plan and propose corresponding monitoring and management strategies, aiming to provide references and guidance for clinicians when formulating treatment plans.

## 2 Case introduction

**Figure 1:**
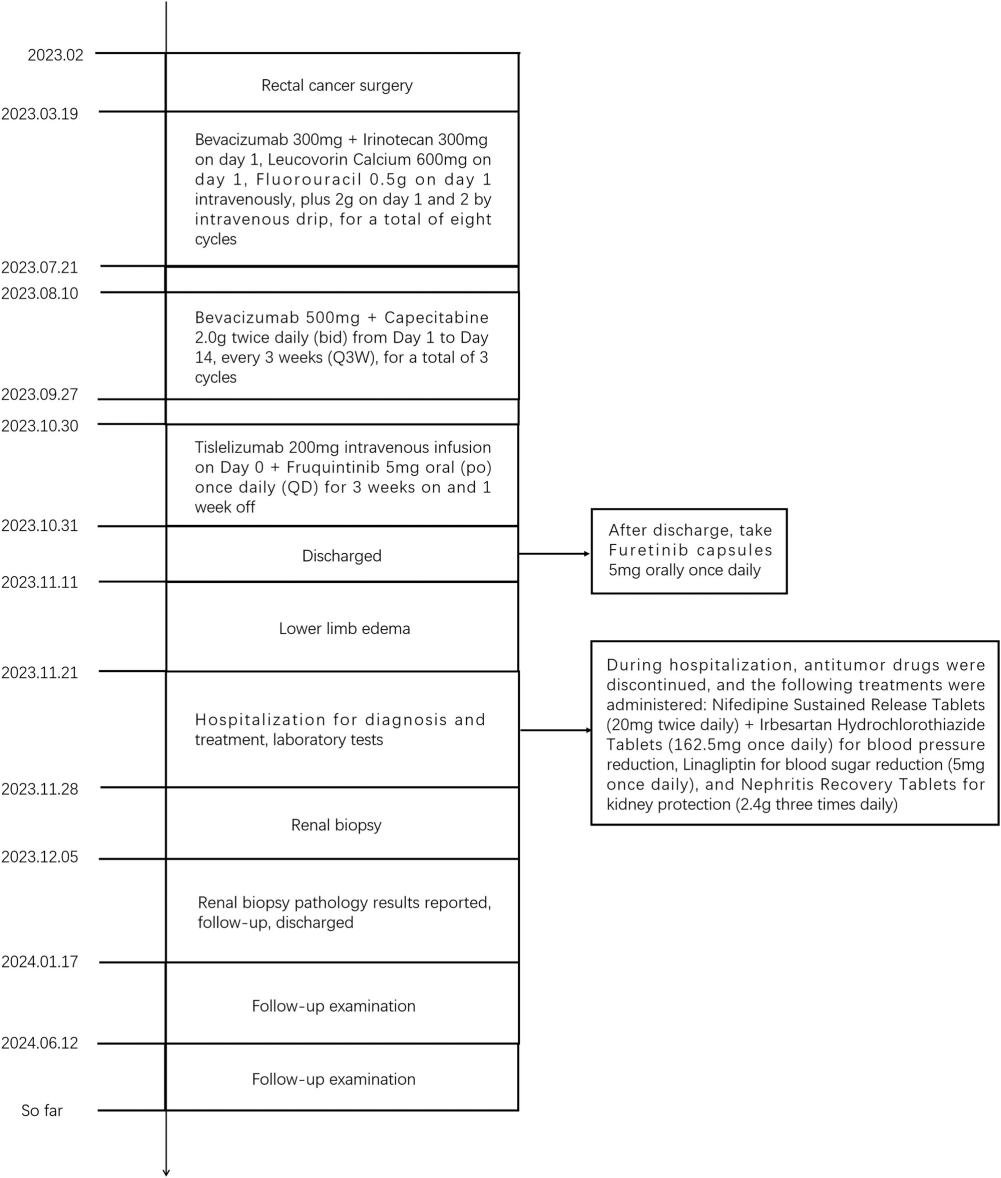
Timeline of major events in the patient’s treatment process.

**Figure 2:**
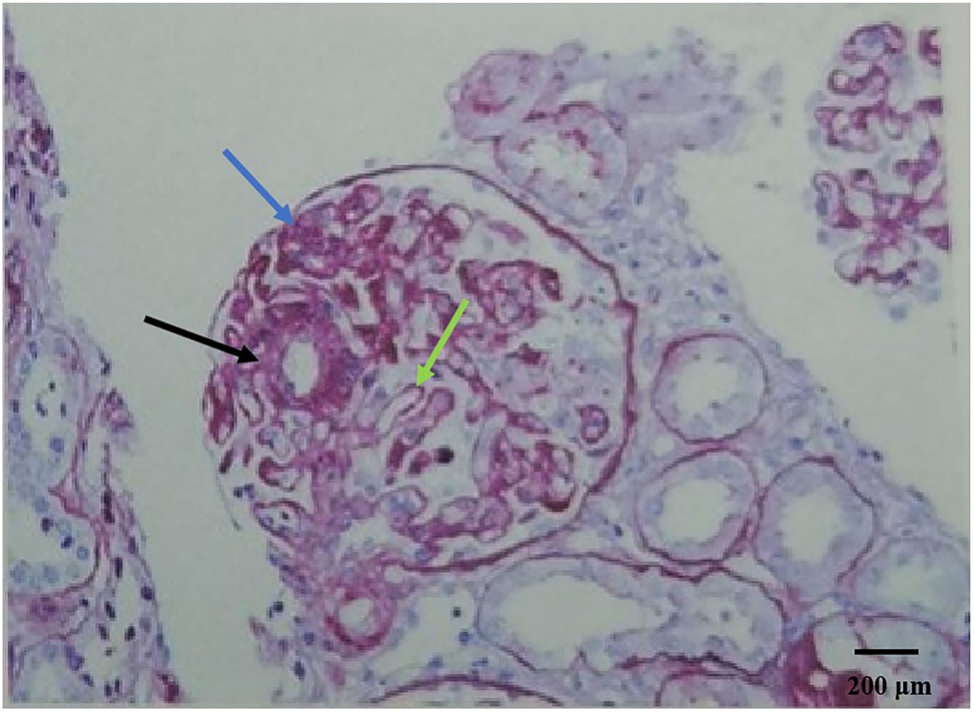
(PAS staining ×200), Black arrows indicate the microvascular tumor-like dilation of the capillary loops, a characteristic feature of glomerular microangiopathy. Blue arrows highlight the areas where there is an increase in the number of cells within the capillaries, suggesting endothelial cell proliferation or swelling. Green arrows point to the segments of the basement membrane that are thickened, with the formation of a double-contour or “tram-track” sign, indicative of basement membrane alterations.

**Figure 3:**
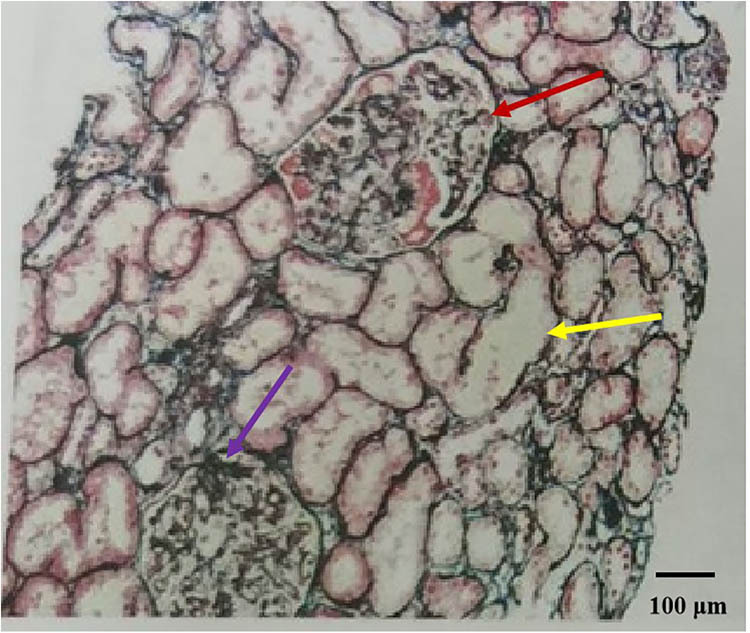
(PASM + Masson ×100), Red arrows delineate areas of suspected small amounts of eosinophilic deposits in the segmental mesangial area and subendothelium, which could be indicative of immune complex deposition. Yellow arrows identify regions where the basement membrane appears denuded, suggesting loss of epithelial cells and exposure of the basement membrane. Purple arrows draw attention to the areas exhibiting features similar to FSGS, including segmental sclerosis and collapse of the capillary loops.

**Figure 4:**
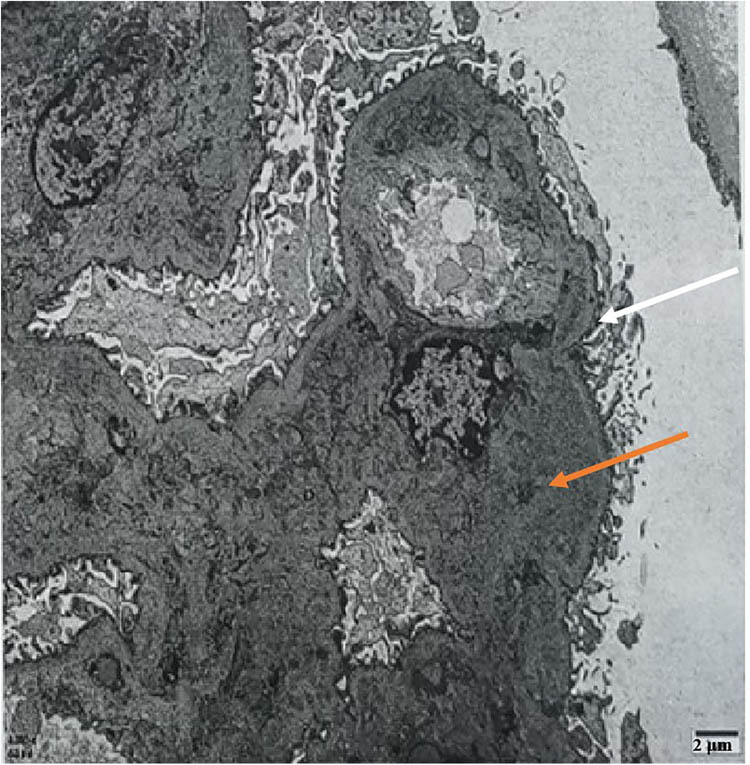
Electron microscope, white arrows indicate segmental mesangial interposition, where mesangial cells or matrix appear to insert into the capillary loop, altering the normal glomerular architecture. Orange arrows point to blocky and clustered electron-dense material in the mesangial area and subendothelial segments, which could represent immune complex deposits or other pathologic accumulations.

The patient, a 60-year-old woman of Chinese Han ethnicity, was admitted on 21 November 2023 after experiencing bilateral lower limb oedema for 10 days. Ten days before admission, the patient experienced symmetric concave oedema of the fingers of both lower limbs without an obvious cause, accompanied by foam urine, without haematuria, and without symptoms of urinary frequency, urgency, or pain. One day before admission, the patient had facial oedema, so she went to the nephrology department of the hospital for treatment. Outpatient laboratory urine routine prompt: occult blood (3+), red blood cell count 26.20/μL, urinary protein (4+) is admitted to the hospital with “proteinuria pending investigation.” She underwent rectal cancer resection in February 2023 and received chemotherapy with bevacizumab (300 mg/course) and fluorouracil regimen starting from 18 March 2023 (a total of eight cycles, with the last medication on 21 July 2023). Evaluation: Stable disease, administered with bevacizumab 500 mg + capecitabine 2.0 g twice a day D1–14 every 3 weeks on 10 August 2023, 31 August 2023 and 27 September 2023, for three cycles of maintenance treatment. Twenty-two days before this visit, the efficacy was evaluated as progressive disease (PD), with a regimen of tislelizumab 200 mg administered intravenously on day 0 + 5 mg fruquintinib taken orally once a day, for a continuous 3-week period followed by a 1-week break from medication. A history of hypertension for more than 10 years, with a maximum of 180/110 mmHg. Regular oral administration of nifedipine sustained-release 40 mg twice a day and irbesartan hydrochlorothiazide tablets 162.5 mg twice a day, with blood pressure (BP) controlled at 130/80 mmHg (Figure 1). The medical history of “diabetes” is >5 months, and “linagliptin 5 mg once a day” is taken orally, which can control blood sugar. Deny medical history of connective tissue disease, cerebrovascular disease, etc. Deny history of recurrent miscarriage and thrombosis, already menopausal. Physical examination: Body temperature 36.8℃, BP: 202/113 mmHg, facial oedema, symmetrical and concave oedema in both lower limbs. Laboratory examination: including routine examination and infection-related indicators, as detailed in Table 1; ultrasound indicates normal renal morphology. After excluding contraindications, a renal biopsy was performed. On 5 December 2023, renal biopsy pathology results: Immunofluorescence: 11G, IgG±, IgA+, IgM+–++, C3+, C1q+, FRA−, Alb+–++, *κ*+, *λ*+, Segmental mesangial area and granular deposition of segmental capillary walls. Microscopic examination revealed 21 glomeruli, 2 ischemic sclerosis, and mild segmental proliferation of mesangial cells and stroma in the remaining glomeruli. Segmental mesangial dissolution, microvascular-like dilation of the capillary loop, diffuse increase in intracellular cells with segmental vacuolisation, thickening of the basement membrane segments, formation of a double track sign, suspicious deposition of a small amount of eosinophil in the segmental mesangial area and subendothelium, with one small cellular crescent formation and two segmental sclerosis accompanied by capillary loop collapse and podocyte proliferation. Vacuolar and granular degeneration of renal tubular epithelial cells, focal shedding of brush hairs, occasional epithelial cell disintegration and bare basement membrane formation, small focal atrophy, and a few protein and epithelial cell tubular types can be seen in the lumen. Renal interstitial small focal lymphocyte and monocyte infiltration with fibrosis. Mild thickening of the small arterial wall with glassy changes, segmental fibrosis and sclerosis of the intima and narrowing of the lumen (Figure 2). Immunohistochemistry: Granular deposition of C4d++ glomerular capillary wall, segmental mesangial area, and segmental small artery wall (Figure 3). Electron microscopy observation: Mild to moderate proliferation of glomerular mesangial cells and matrix, segmental mesangial insertion, blocky and clustered electron-dense material in the mesangial area and subendothelial segments, with most of the epithelial foot processes fused. Vacuolar degeneration and increased lysosomes in renal tubular epithelial cells (Figure 4). There is no obvious lesion in the renal interstitium. Pathological diagnosis: (1) GMA with focal segmental glomerulosclerosis (FSGS)-like lesions, combined with clinical considerations, is drug-related renal injury; and (2) immune complex-mediated glomerulonephritis.

**Table 1:** Laboratory inspection results

Item	Result	Reference range
24-h urine protein quantification	16.15 g/day	≤0.15 g/day
Albumin	28 g/L	35–51 g/L
Lactate dehydrogenase	449 U/L	100–300 U/L
Platelet	84 × 10^9^/L	100–300 × 10^9^/L
Creatinine	104 μmol/L	70–106 μmol/L
Immunoglobulin IgG	5.41 g/L	7.0–17.0 g/L
Antic-phospholipase A2 receptor antibody	1.82 RU/mL	＜1.0 RU/mL
Rheumatoid factor negative	Negative	—
Respiratory tract infections	Negative	—
Syphilis-AIDS antibody	Negative	—
Vasculitis antibody	Negative	—
Immunofixation electrophoresis of blood and urine	Negative	—

While hospitalised, the patient discontinued the use of anti-tumour drugs and initiated treatment, which included nifedipine sustained-release tablets (40 mg twice a day, at 8:00 and 20:00) for BP management, irbesartan hydrochlorothiazide tablets (162.5 mg once a day, at 20:00), linagliptin (5 mg once a day, at 12:00) for glycaemic control, and Nephritis Rehabilitation Tablets (2.4 g three times a day, at 9:00, 14:00, and 19:00) for renal protection. Considering the protection of the liver, the patient did not receive glucocorticoids and was discharged with medication. At discharge, oedema in both lower limbs was reduced. There was a recheck on 17 January 2024, and the detailed results are shown in Table 2. After a 6-month follow-up, the patient’s kidney condition had recovered well. However, the wall of the rectum and sigmoid colon had thickened, some nodules in both lungs had increased in size, and the pelvic lymph nodes had slightly enlarged; the efficacy was evaluated as PD.

**Table 2:** Comparison of results between patients discharged from the hospital and follow-up 2 months later

Item	Discharge	Review after 2 months	Reference range
Urine protein	**+++**	**+**	—
24-h urine protein quantification	2.19 g/day	0.96 g/day	≤0.15 g/day
Platelet	152 × 10^9^/L	245 × 10^9^/L	100–300 × 10^9^/L
Albumin	29 g/L	38 g/L	35–51 g/L
Creatinine	72 μmol/L	59 μmol/L	70–106 μmol/L


**Informed consent:** Informed consent has been obtained from all individuals included in this study.
**Ethical approval:** The research related to human use has been complied with all the relevant national regulations, institutional policies and in accordance with the tenets of the Helsinki Declaration, and has been approved by the Ethics Committee Affiliated Hospital of HeBei University, approval number HDFYLL-KY-2024-146.

## 3 Discussion

The patient had previously received anti-tumour drug treatment, including bevacizumab, irinotecan, fluorouracil, and capecitabine. During the follow-up period, there were no signs of proteinuria or haematuria, and renal function remained within the normal range. Due to disease progression, tislelizumab and fruquintinib were incorporated into the treatment regimen. About 10 days after the initiation of treatment, the patient developed microvascular disease with FSGS-like lesions, as well as immune complex-mediated glomerulonephritis. Given the temporal relationship and direct association with the use of tislelizumab and fruquintinib, the patient’s renal dysfunction is attributed to GMA induced by one or both of these agents.

Thrombotic microangiopathies are a series of diseases characterised by endothelial damage leading to microvascular thrombosis and ischaemia [11]. Many factors can cause endothelial damage, such as pregnancy, infection, malignant hypertension, medication, malignant tumours, and autoimmune diseases [12]. Drug-induced TMA is a rare but potentially fatal complication. It usually manifests as a systemic disease accompanied by a classic triad of haemolytic anaemia, thrombocytopenia, and organ damage [11]. Angiotensin-converting enzyme inhibitors are the main therapeutic drugs for renal-restrictive TMA. However, in cases with a large amount of proteinuria within the nephrotic syndrome spectrum, the efficacy is limited [13]. In this case, after discontinuing tislelizumab and fruquintinib, an increase in serum albumin, a decrease in urine protein, normalisation of serum creatinine, relief of oedema, and an improvement in renal function were observed during a 6-month follow-up.

It is worth noting that compared with other VEGFR inhibitors, fruquintinib exhibits pharmacological efficacy at lower blood concentrations, thereby improving safety [14]. However, similar to other VEGFR inhibitors, hypertension, and proteinuria are common adverse reactions to fruquintinib. In our case, the patient developed proteinuria after the use of fruquintinib, which is similar to the case reported by Zhao et al. [13]. This emphasises the importance of monitoring the patient’s renal function when using anti-VEGF drugs in clinical practice. As pointed out by Zhao et al., the use of fruquintinib is associated with the occurrence of kidney-restricted TMA, which requires us to remain vigilant during treatment and adjust the treatment plan in time when necessary. In addition, their study also highlights the importance of renal biopsy when proteinuria occurs for early diagnosis and treatment of potential TMA. Our case further confirms that fruquintinib may be associated with the occurrence of renal TMA and emphasises the consideration of renal safety in cancer treatment. The combination of VEGF inhibitors with immune checkpoint inhibitors (ICIs) demonstrates significant synergistic effects in anti-tumour therapy. On one hand, it reduces the blood supply to tumours through anti-pathogenesis, and on the other hand, it enhances the infiltration of T cells into tumours by blocking tumour-induced immune suppressive cells, thereby increasing the anti-tumour activity of ICIs [15]. This joint strategy not only improves the immunosuppression state in the tumour microenvironment but also promotes the aggregation and activation of immune cells through vascular normalisation, forming a positive feedback loop that could theoretically lead to more durable therapeutic effects [16]. However, numerous reports indicate an increasing risk of renal complications associated with ICIs in clinical use, with mechanisms including autoantibody-induced autoimmune reactions against renal tubular epithelial cells, “off-target effects” from ICIs binding to checkpoint receptors expressed in the kidney, and the promotion of activation and proliferation of specific T cells by ICIs [2]. Consequently, this combined medication also presents new challenges, especially regarding renal safety. VEGF inhibitors may directly affect kidney function by inhibiting the VEGF signalling pathway, while ICIs might indirectly impact the kidneys by enhancing immune responses [17]. The combined use of these two types of drugs could have a synergistic effect, increasing the risk of kidney damage, manifested as hypertension, proteinuria, and other adverse reactions, complicating the diagnosis and treatment of toxicity. Therefore, when implementing this combined therapy, it is essential to strictly monitor and manage the patient’s renal function to ensure the safety and efficacy of the treatment. Horino et al. [18] reported a striking case involving a patient with hepatocellular carcinoma who developed TMA after treatment with atezolizumab, a PD-L1 blocker, and bevacizumab, an inhibitor of VEGF. This case particularly emphasises the potential for exacerbated kidney-related side effects when combining new classes of antineoplastic drugs, indicating the need for caution in treatment planning and vigilant monitoring of the patient’s kidney function. Although this combined treatment strategy theoretically offers synergistically enhanced anti-tumour effects, it also presents new challenges, particularly in managing the renal toxicity associated with these drugs. Therefore, for patients undergoing such combination therapies, we must remain highly vigilant, promptly recognise any adverse renal reactions and adjust treatment strategies as necessary to ensure patient safety and therapeutic efficacy. Moreover, this case reminds us that in cancer treatment, it is essential to consider both the efficacy of the drugs and their potential side effects comprehensively to achieve personalised treatment and optimal patient care.

Anti-tumour drugs have a certain impact on renal function in clinical treatment, and the pathogenesis and pathological manifestations of renal damage caused by different anti-tumour drugs vary [19]. When anti-VEGF drugs are used in combination with other anti-cancer drugs, special attention must be paid to the potential exacerbation of adverse renal reactions. These drugs may affect kidney function through various mechanisms, leading to proteinuria, hypertension, and other adverse events. Therefore, understanding the normal role of the VEGF signalling pathway and the side effects that may arise from the low target selectivity of these drugs is crucial when using these combined therapies. For patients receiving anti-VEGF drug treatment, regular monitoring of proteinuria and BP is essential to detect potential kidney issues at an early stage. If symptoms occur or worsen, medication should be stopped immediately, and a timely renal biopsy should be performed, if possible, to facilitate early diagnosis of TMA. Furthermore, fostering communication and collaboration among experts in oncology, nephrology, and pharmacy is crucial for the early identification and management of kidney toxicity induced by anti-cancer drugs. This interdisciplinary approach aids in timely intervention, improving the prognosis of patients with malignant tumours and enhancing their quality of life. Through this comprehensive management strategy, we can better balance efficacy and safety in cancer treatment, ensuring that patients experience the best possible treatment outcomes.

**Limitations**

1. Although it is speculated that tislelizumab or fruquintinib may lead to renal restrictive TMA, there is a lack of further mechanistic research to confirm this association.
2. Patients have multiple potential influencing factors, such as the progression of the tumour itself and the history of other drug use. These factors may affect the progression of the disease and treatment effectiveness.
3. Without comparative data with other patients or different treatment plans, it is impossible to confirm whether the observed phenomenon is universal or only an isolated case.

Due to this being a standalone case study, there may be selective biases in selection and reporting, which can affect the broad applicability of the conclusions.

These limitations affect, to some extent, the universality and reliability of the research results, which need to be validated and expanded through larger-scale studies and longer follow-up periods in the future.

## 4 Conclusion

The results of this study suggest that tislelizumab or fruquintinib may lead to renal restrictive TMA, a rare but life-threatening complication of cancer treatment drugs. Therefore, for all patients receiving anti-VEGF drug treatment, regular monitoring of proteinuria and BP is necessary. Kidney biopsy should be performed promptly to detect thrombotic microvascular disease early.
